# Hormone therapy and psychosocial factors in menopausal women with painful TMD: a pilot cross-sectional study

**DOI:** 10.1590/1678-7765-2025-0586

**Published:** 2026-04-28

**Authors:** Lays Nóbrega Gomes Farias, Raimundo Euzébio da Costa, Mayara Abreu Pinheiro, Yuri Wanderley Cavalcanti

**Affiliations:** Universidade Federal da Paraíba Departamento de Clínica e Odontologia Social João Pessoa Paraíba Brasil Universidade Federal da Paraíba, Departamento de Clínica e Odontologia Social, João Pessoa, Paraíba, Brasil.

**Keywords:** Menopause, Temporomandibular joint dysfunction syndrome, Psychosocial effects of the disease

## Abstract

**Introduction:**

Temporomandibular Disorder (TMD) is a musculoskeletal condition prevalent in women, especially during menopause. This phase of life is marked by decreased estrogen levels, which can exacerbate pain and psychosocial symptoms.

**Objective:**

This study aimed to evaluate the association between menopause with and without hormone replacement therapy, grade of chronic pain, jaw functional limitations, and symptoms of anxiety and depression in women with painful TMD.

**Methodology:**

This exploratory cross-sectional study was conducted with 45 women diagnosed with painful TMD, divided into three groups: women with regular menstrual cycles (RMC, n=15), women in menopause without hormone replacement therapy (M, n=14), and women in menopause with hormone replacement therapy (MWH, n=16). The evaluation was conducted using the following instruments: the Graded Chronic Pain Scale (GCPS-2), the Patient Health Questionnaire (PHQ-9), the Generalized Anxiety Disorder Scale (GAD-7), the Jaw Functional Limitation Scale (JFLS-8), and the Pain Catastrophizing Scale (B-PCS). The data were analyzed descriptively using Jamovi software (version 2.3.21). Analysis of variance (ANOVA) was performed, followed by multivariate analysis of covariance (MANOVA). Tukey's post-hoc test was used to identify differences between groups. The significance level adopted for all analyses was 5%.

**Results:**

The mean age of participants was 50.0 years (±13.7). The overlap between muscle and joint disorders was most prevalent among the groups. The interference score was significantly lower in the MWH group (p=0.003), as was also the case with depressive symptoms (p<0.001) and anxious symptoms (p=0.012).

**Conclusion:**

The findings highlight the association between menopause, especially with hormone replacement therapy, and emotional and psychosocial aspects related to pain, with lower scores for interference with daily activities, depression, anxiety, and fewer catastrophic thoughts about pain.

## Graphical abstract

**Figure:**
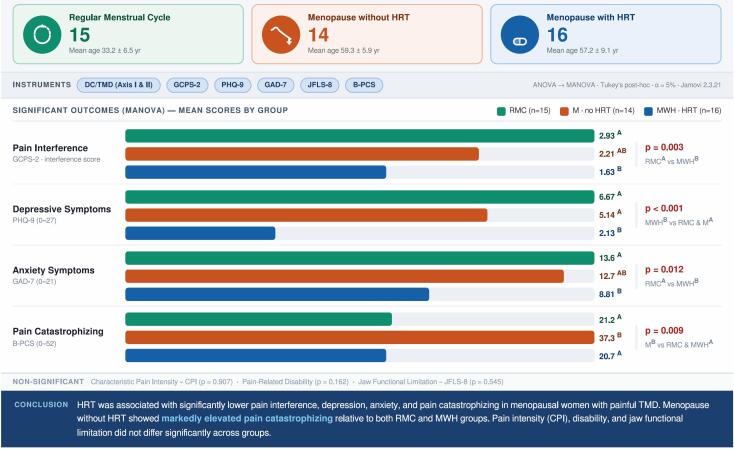
This manuscript is derived from a doctoral thesis and includes data and analyses that were previously presented and discussed in an academic context, with modifications and adaptations made to comply with the format and standards of scientific publication.

## Introduction

Temporomandibular disorder (TMD) is a musculoskeletal condition characterized by pain and/or dysfunction of the temporomandibular joint (TMJ), the masticatory muscles, and associated structures.^1^ For the diagnosis of TMDs, the Diagnostic Criteria for Temporomandibular Disorders (DC/TMD) is the most widely used and disseminated methodology in the literature. This approach includes 12 possible TMD diagnoses, encompassing both painful conditions—such as myalgia, arthralgia, and headache attributed to TMD—and non-painful conditions, such as disc displacements, degenerative joint disease, and subluxation.^2,3^

Pain resulting from TMD may be associated with reduced quality of life, functional limitations, and altered psychosocial characteristics, including anxiety, depression, and sleep disorders.^4–6^ Its prevalence is estimated to be higher in women, particularly during specific life stages, such as menopause.^7,8^

In this context, the menopausal period is marked by a decline in estrogen levels, a hormone known for its influence on pain modulation and the central nervous system.^9,10^ The relationship between this hormone and pain can be explained via the trigeminal pathway, given the presence of estrogen receptors in neuronal and glial cells of the trigeminal spinal nucleus, supporting the hypothesis that estrogen may directly influence the excitability of trigeminal neurons in humans.^11^ Thus, evidence suggests that this hormonal transition increases the risk of chronic pain and may exacerbate painful conditions—particularly musculoskeletal and joint-related disorders—as well as worsen associated psychosocial symptoms.^12–14^

Studies indicate that menopause increases the risk of developing symptoms of anxiety and depression.^13,14^ The vasomotor symptoms characteristic of this period, such as hot flashes and night sweats, have often been associated with these disorders, although the underlying mechanisms have not yet been fully clarified.^13–17^ Given the multifactorial nature of these psychosocial disorders, one suggested aspect is that fluctuations in estrogen levels may increase vulnerability to anxiety and depression, as this hormone affects the serotonin and gamma-aminobutyric acid (GABA) signaling pathways.^14^

In addition to biological factors, psychological and social characteristics also need to be investigated as contributors to anxiety and depression; however, the literature on these aspects remains scarce. In this context, it is known that neuroticism—defined as a personality trait characterized by a tendency to experience negative emotions more frequently and intensely—increases women's vulnerability to depression during menopause.^18^ The analysis of pain catastrophizing would complement this finding, as neuroticism reflects the emotional dimension, while catastrophizing would provide additional insights regarding cognitive processes. Pain catastrophizing is defined as the presence of excessively negative and debilitating thoughts about the pain experience and is considered a relevant psychological factor that amplifies pain perception and is associated with poorer prognoses in various chronic pain conditions.^19^

Greater pain catastrophizing is associated with a more intense perception of pain and a decrease in endogenous inhibitory controls, such as the Conditioned Pain Inhibition (CPI) phenomenon.^20^ Although the impact of pain catastrophizing has been extensively studied in different phases of the menstrual cycle and in women with endometriosis, there is a paucity of evidence exploring the relationship between pain catastrophizing and menopause.^21,22^

Considering that menopause is associated with a decline in quality of life and an increase in the prevalence of temporomandibular disorders (TMD), despite limited evidence of functional limitations related to this condition, and recognizing that menopause is also linked to the worsening of psychological conditions, particularly psychosocial disorders, this study aimed to evaluate the association between menopause with and without hormone replacement therapy and the grade of chronic pain, jaw functional limitations, and symptoms of anxiety and depression in women with painful TMD. The null hypothesis of this study is that menopausal women without hormone replacement therapy do not show differences in pain intensity, functional limitations, and levels of anxiety and depression when compared to those using hormone replacement therapy and women with a regular menstrual cycle.

## Methodology

This exploratory cross-sectional study was conducted at the teaching clinic of Faculdades Integradas de Patos, in Campina Grande, Paraíba, Brazil. The study included 45 women with painful TMD who were registered as patients at the clinic. The women were conveniently distributed into three groups: women with a regular menstrual cycle (RMC = 15), women in menopause without hormone replacement therapy (M = 14), and women in menopause with hormone replacement therapy (MWH = 16). For inclusion in the menopause groups, a prior medical diagnosis and the absence of menstruation for a minimum period of one year were required. To validate the methodology and define the sample, a pilot study was initially conducted with 10 women, who were incorporated into the study. The sample size was estimated with a power of β = 0.8 and α = 0.05. A minimum of 14 patients per group was established for this study. The Ethics Committee of Faculdades Integradas de Patos, in Campina Grande, Paraíba, Brazil approved this study (protocol no. 67896023.6.0000.5181).

Women without natural teeth were excluded from the study due to the loss of occlusal stability; women with partial loss of natural teeth compromising occlusal stability, regardless of the number of missing teeth, were also excluded. Additionally, the following were considered exclusion criteria: the presence of cognitive disorders that affected the comprehension of the questionnaires; odontogenic pain; undergoing orthodontic treatment; undergoing treatment for TMD; medical conditions with a negative impact on the central nervous system, such as cancer, brain or spinal cord injuries, neurological diseases, or multiple sclerosis; pregnancy; use of centrally acting analgesics in the past 24 hours; history of drug use; and uncontrolled diabetes, lupus, leprosy, hypertension, or postsurgical hypoesthesia.^23,24^ Women who were in their menstrual period at the time of the tests were not included.^23,24^

Initially, all participants underwent an interview to confirm the inclusion criteria. The diagnosis of painful TMD was made by a single trained examiner using the criteria established by the Diagnostic Criteria for Temporomandibular Disorders (DC/TMD). This instrument consists of two axes of evaluation, with Axis I encompassing the physical examination that guides the clinical diagnosis of TMD and incorporates the three major diagnostic categories: muscular TMD, articular TMD, and headache attributed to TMD.^25,26^ While Axis II incorporates already validated instruments that enable the analysis of pain intensity, psychosocial distress, and pain-related disability. In this study, the following assessment tools were utilized: the Graded Chronic Pain Scale, version 2 (GCPS-2); the Patient Health Questionnaire (PHQ-9); the Generalized Anxiety Disorder Scale (GAD-7); and the Jaw Functional Limitation Scale (JFLS-8).^25,26^ In addition to these instruments, the Pain Catastrophizing Scale (B-PCS) was utilized to assess the degree of pain catastrophizing among participants.

The GCPS-2 includes three items that assess pain intensity on a scale of 0 to 10: current pain, worst pain in the past 30 days, and average pain in the past 30 days. Additionally, three items evaluate the impact of pain on an individual's life, including interference with daily activities, recreational, social, and family engagements, as well as work ability. Finally, one item records the number of days when pain prevented the individual from carrying out normal activities, such as working, studying, or performing household chores. Based on these data, three variables were generated: the characteristic pain intensity (CPI), obtained by averaging the three intensity items and multiplying by 10 (resulting in a score from 0 to 100), which indicates the intensity of the pain experienced; the interference score, calculated from the average of items six to eight and multiplied by 10, which reflects the impact of pain on daily activities, social activities, and work activities; and the disability associated with pain, which is calculated from the number of days pain prevented the performance of daily activities, as well as the disability score, reflecting how much pain interferes with daily, leisure, and work activities.^27^

The PHQ-9 comprises nine items designed to assess depressive symptoms, while the GAD-7 includes seven items for evaluating anxiety symptoms. Each item is scored on a Likert-type scale, ranging from 0 (never) to 3 (almost every day).^28^ The sum of scores generates a total in which scores of 5, 10, and 15 represent the cut-off points for mild, moderate, and severe conditions, respectively.^28^

The Jaw Functional Limitation Scale (JFLS) was developed to assess the overall functional limitations of the masticatory system, consisting of items that evaluate chewing, jaw mobility, and both verbal and non-verbal communication.^29^ The items are scored on a scale ranging from 0 (no limitation) to 10 (severe limitation) using a Likert-type scale. A general score is calculated based on these items.^29^

The aspects related to pain catastrophizing were assessed using the Pain Catastrophizing Thoughts Scale (B-PCS), translated and validated by Sardá Junior, et al.^30^ (2008). The B-PCS consists of 13 statements that describe thoughts and feelings related to painful experiences, with the degree to which these thoughts and feelings occur during pain being rated on a scale from 0 to 4. The total score, ranging from 0 to 52, is obtained by summing all items. Additionally, three subscale scores assess rumination, magnification, and hopelessness.^30^ In this study, the general score was used.

The data were tabulated and analyzed descriptively using Jamovi software (2.3.21). Initially, the data were analyzed using analysis of variance (ANOVA) to assess the differences in the outcome variables: characteristic pain intensity, interference score, pain-associated disability, symptoms of depression, symptoms of anxiety, jaw functional limitation score, and pain catastrophizing. For variables showing a statistically significant association, a multivariate analysis of covariance (MANOVA) was conducted to provide a more comprehensive evaluation of the relationships among the studied factors. Tukey's post-hoc test was applied to determine differences between groups. A significance level of 5% was set for all analyses.

## Results

The women included in the study had a mean age of 33.2 ± 6.51 years in the RMC group, 59.3 ± 5.93 years in the M group, and 57.2 ± 9.06 years in the MWH group, were predominantly Brown (n=22), had completed high school (n=26), and were married (n=21) (Table 1). Regarding the diagnosis of TMD, muscle and joint disorders were found to occur concomitantly with greater frequency in menopausal women both with (n=8) and without hormone replacement therapy (n=10), as well as in the control group (n=13) (Table 2).

**Table 1:** Sample characterization according to race, educational level, and marital status

	RMC (n=15)		M (n=14)		MWH (n=16)	
	n	%	n	%	n	%
**Race**						
White	6	33.3	3	16.7	9	50.0
Black	2	40.0	2	40.0	1	20.0
Brown	7	31.8	9	40.9	6	27.3
**Educational level**						
Incomplete primary education	0	0	0	0	3	100
Complete primary education	0	0	3	60.0	2	40.0
Complete secondary education	9	34.6	8	30.8	9	34.6
Complete higher education	6	54.5	3	27.3	2	18.2
**Marital status**						
Single	6	40.0	2	13.3	7	46.7
Married	7	33.3	9	42.9	5	23.8
Divorced	2	40.0	2	40.0	1	20.0
Widowed	0	0	1	25.0	3	75.0
						

RMC (Regular menstrual cycle), M (Menopause without hormone replacement therapy), MWH (Menopause with hormone replacement therapy)

**Table 2:** Distribution of Temporomandibular Disorder (TMD) diagnoses in women with regular menstrual cycle

	RMC (n=15)		M (n=14)		MWH (n=16)			
	n	%	n	%	n	%	n	%
Muscular only	2	15.4	4	30.8	7	53.8	13	100.0
Joint only	0	0	0	0	1	100	1	100.0
Muscle and joint	13	41.9	10	32.2	8	25.9	31	100.0

RMC (Regular menstrual cycle), M (Menopause without hormone replacement therapy), MWH (Menopause with hormone replacement therapy)

**Table 3:** Mean scores of psychosocial factors according to menopausal status, with or without hormone replacement therapy

	RMC (n=15)	M (n=14)	MWH (n=16)	p†	p*
Characteristic Pain Intensity (CPI)	74.4±23.5	71.4±29.5	76.0±26.5	0.907	
Interference score	2.93±2.8^A^	2.21±0.55A,B	1.63±0.64^B^	<0.001	0.003
Pain-related disability	5.27±0.79	4.93±1.38	4.56±1.15	0.162	
Depression symptoms	6.67±3.79^A^	5.14±1.88^A^	2.13±1.71^B^	<0.001	<0.001
Anxiety symptoms	13.6±5.6^A^	12.7±3.79A,B	8.81±4.46^B^	0.019	0.012
Mandibular movement limitation score	2.47±1.81	1.79±1.57	1.93±1.57	0.545	
Pain catastrophizing	21.2±16.6^A^	37.3±19.8^B^	20.7±7.28^A^	0.026	0.009

RMC (Regular menstrual cycle), M (Menopause without hormone replacement therapy), MWH (Menopause with hormone replacement therapy)

† One-way ANOVA test

* MANOVA test.

A,B Tukey test, different letters indicate statistically significant differences (<0,05).

The analysis of psychophysical measurements revealed that, among the variables derived from the GCPS-2, only the interference score showed a statistically significant difference between the RCM and MWH groups (p < 0.001). Although there was no significant difference in depressive symptom scores between the RCM and M groups, both groups had significantly higher scores than the hormone replacement therapy (HRT) group (p < 0.001). Regarding anxiety symptoms, scores were similar between the M and MWH groups, as well as between the RCM and M groups; however, a statistically significant difference was observed between the RCM and MWH groups (p = 0.012). Pain catastrophizing was significantly higher in the M group compared to the other groups (p = 0.009). Finally, no significant differences were found in mandibular movement limitations among the evaluated groups.

## Discussion

The findings of this pilot and exploratory study provide new evidence on the influence of menopause on the psychosocial aspects of women with painful TMD. Among the studied groups, overlapping muscular and joint diagnoses were more prevalent. Literature regarding prevalence data for this specific population is scarce. However, joint disorders are known to be more prevalent in women with infertility and hormonal disorders, which contrasts with the findings of this study.^31^

Regarding the variables generated by the GCPS-2, characteristic pain intensity and pain-related disability were similar among the groups studied. However, the interference score showed a statistically significant difference between the RMC and MWH groups. This variable assesses the extent to which pain interferes with participants' daily activities, with the lowest score—indicating the least interference—observed in the MWH group. One factor that may help explain this result is that hormone replacement therapy improves vasomotor symptoms, which are associated with reduced quality of life in menopausal women.^32,33^ Furthermore, for these women, hormone therapy can also improve productivity at work.^34^ Another factor to consider is the age range of groups. Menopausal women are older, which may reduce the burden and workload of household tasks compared to younger women, who often juggle multiple roles, balancing work activities with caring for the home and children.^35^

Moreover, evidence suggests that menopause is a risk factor for the development of anxiety and depression symptoms.^13,14^ The significantly lower scores for depressive symptoms in the MWH group, compared to the RMC and M groups, reinforce the hypothesis that hormone replacement therapy may play an important role in improving the psychological well-being of these women. These findings are consistent with the results of Shea, et al.^33^ (2020), who observed that hormone therapy may facilitate the reduction of more severe vasomotor symptoms, which are considered a risk factor for the development of depressive symptoms.^33^ However, the results pertain to early menopause, suggesting that menopause duration is an important variable that should be considered and evaluated in the context of symptom development.

Anxiety scores were also lower in the MWH group, which contrasts with the results of Feng, et al.^36^ (2022), who suggest that hormone therapy does not significantly affect anxiety regulation, despite its known effectiveness in improving depressive symptoms in postmenopausal women.^36^ However, it is important to note that Feng et al.'s results apply to overweight women, a condition that has been independently associated with anxiety in some studies.^37,38^ The mean JFLS scores for mandibular movements were similar across all three evaluated groups. This finding suggests that jaw functional capacity remained comparable, regardless of each group's specific conditions or characteristics.

Beyond psychosocial factors, pain catastrophizing is a psychological construct consistently associated with worse pain outcomes in women²². Although no studies have directly clarified whether catastrophizing modifies pain levels specifically in menopausal women, evidence from other female hormonal conditions, such as dysmenorrhea, demonstrates that catastrophizing is associated with increased pain intensity.^39^ Moreover, available data indicate that heightened pain may, in turn, exacerbate catastrophizing, suggesting a bidirectional relationship between these phenomena.^40^ Considering that menopause is characterized by significant hormonal fluctuations capable of influencing cognitive–emotional pain processing, it is plausible that this life stage may affect catastrophic thinking patterns, particularly in the absence of hormone replacement therapy. In this study, catastrophizing scores were significantly lower in the hormone replacement therapy group, suggesting that HRT may exert a protective effect by reducing pain interference in daily activities and attenuating catastrophic thoughts that negatively influence pain-related behaviors. The scarcity of studies specifically evaluating pain catastrophizing in menopausal women reinforces the relevance and originality of the present findings.

In summary, the findings of this study contribute to advancing the understanding of the relationship between menopause and psychosocial aspects in women with painful TMD. However, certain methodological limitations should be considered when interpreting the results. A convenience sampling strategy was adopted due to the inherent challenges in recruiting a specific clinical population meeting well-defined diagnostic criteria, which may limit the external generalizability of the findings. Although this approach is common in exploratory clinical studies, the possibility of selection bias must be acknowledged.

Furthermore, it was not possible to standardize menopausal stage (i.e., time since the last menstrual period) or the type, dosage, and duration of hormone replacement therapy, variables that may influence psychosocial outcomes and pain perception. The lack of control over these factors precludes a more precise estimation of the specific effects of hormone therapy, and the results should therefore be interpreted within this context. Despite these limitations, the groups were rigorously defined based on standardized clinical diagnostic criteria (DC/TMD) and validated psychometric instruments, which strengthen the internal consistency and methodological rigor of the analyses performed.

Future research should incorporate longitudinal monitoring to enable a more comprehensive assessment of the influence of menopause duration and various hormonal therapies on clinical and psychosocial outcomes. Additionally, measuring serum estrogen hormone levels in the blood of the evaluated women would provide valuable supplementary data.

## Conclusion

The findings of this study suggest that menopause is linked to significant emotional and psychosocial changes related to pain in women with painful TMD. Specifically, menopausal women undergoing hormone replacement therapy had lower scores for pain interference in daily activities, along with reduced levels of depression, anxiety, and catastrophic thoughts about pain.

## Data Availability

All data generated or analyzed during this study are included in this published article.
